# Role of single-use gastroscopes in advanced endoscopy

**DOI:** 10.1016/j.vgie.2023.11.006

**Published:** 2023-11-22

**Authors:** Francisco Baldaque-Silva, João Pedro Pereira, Miroslav Vujasinovic, Naining Wang, Masami Omae

**Affiliations:** 1 Division of Medicine, Department of Upper Gastrointestinal Diseases, Karolinska University Hospital and Karolinska Institute, Stockholm, Sweden; 2 Advanced Endoscopy Center Carlos Moreira da Silva, Department of Gastroenterology, Pedro Hispano Hospital, Matosinhos, Portugal; 3 Department of Pathology, Karolinska University Hospital and Karolinska Institute, Stockholm, Sweden

## Introduction

Recent studies have highlighted the risk of endoscopy-associated infections.^1^, ^2^, ^3^, ^4^ Infections are more frequent in ERCP and EUS^2^^,^^5^; however, even non-ERCP/EUS GI endoscopic procedures may be associated with infection in 0.12% of cases.^2^ Endoscopy-associated infections can be life threatening in specific clinical conditions such as in immune-compromised patients and in prion infections.^3^

The increased costs associated with reprocessing, storage, and maintenance of endoscopes and the lack of dedicated personnel, especially during night shifts and weekends, create logistical and financial pressure in most endoscopy units.^4^^,^^6^ There is also an environmental impact associated with the use of reprocessed endoscopes.^7^

To overcome the risks of infection and these logistic pressures, single-use platforms have been developed.^1^^,^^8^ However, their use is controversial primarily because of their environmental impact, weighing against potential reduction in endoscopy-associated infection rates.^2^ Recent developments on plastic enzymatic degradation offer a potentially green and scalable route for polyester-waste recycling and might increase the value of single-use endoscopes in current clinical practice.^9^ If these endoscopes can be used in diagnostic and therapeutic procedures and if their environmental impact decreases, their role in clinical practice may increase in the future.

**Figure 1:**
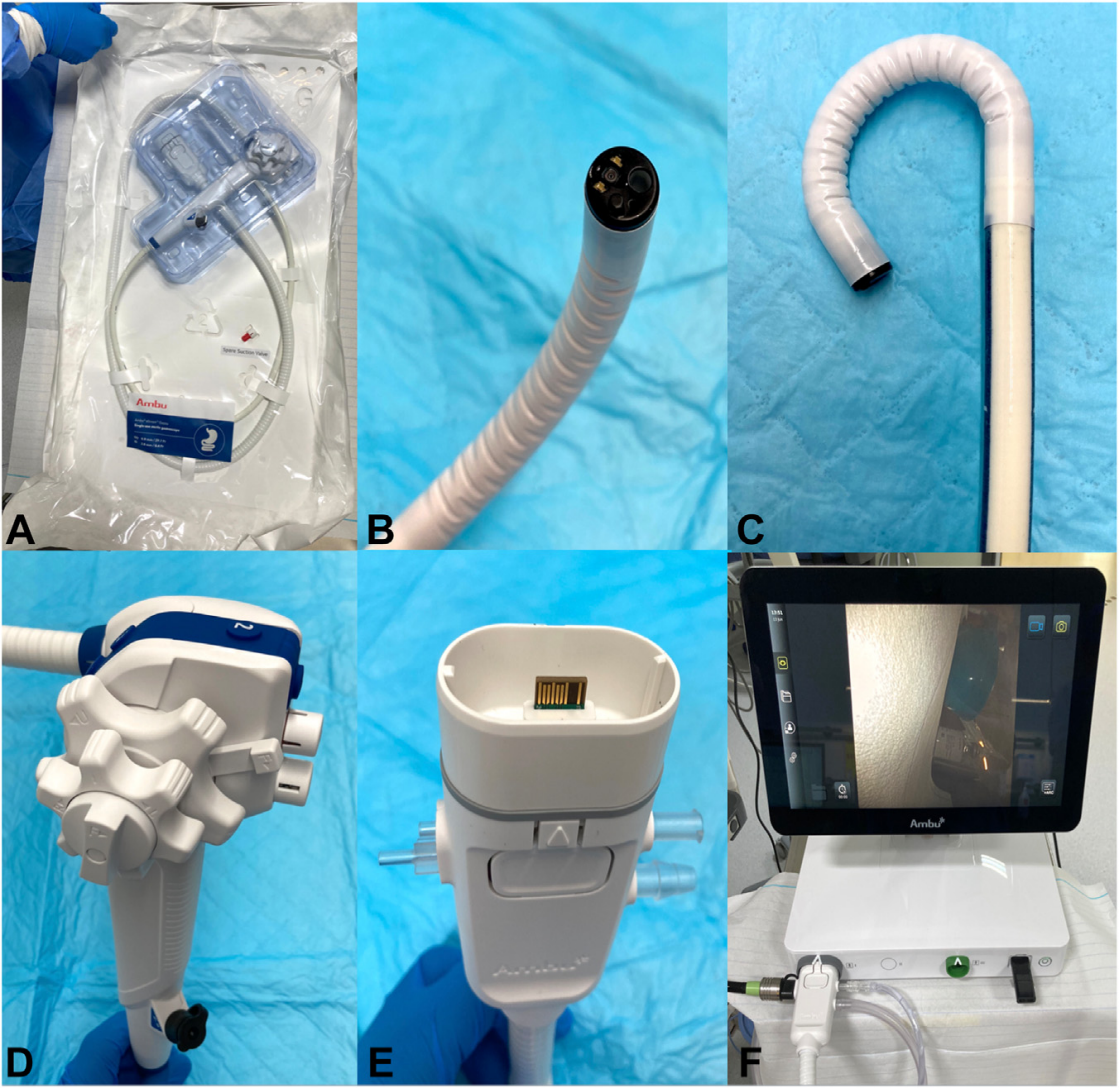
**A,** The aScope (Ambu, Ballerup, Denmark) is a single-use and sterile endoscope. **B,** The distal tip has an outer diameter of 9.9 mm, dual LEDs, 140° field of vision, a 2.8-mm working channel, and a water jet. **C,** Capacity of retroflection is up to 210°. **D,** As with conventional endoscopes, there are 2 wheels, 2 locks, and several configurable buttons. **E,** The scope has a connector that is compatible with standard ancillary devices and tube sets. **F,** This connector connects to a small processor with light source, and a screen.

**Figure 2:**
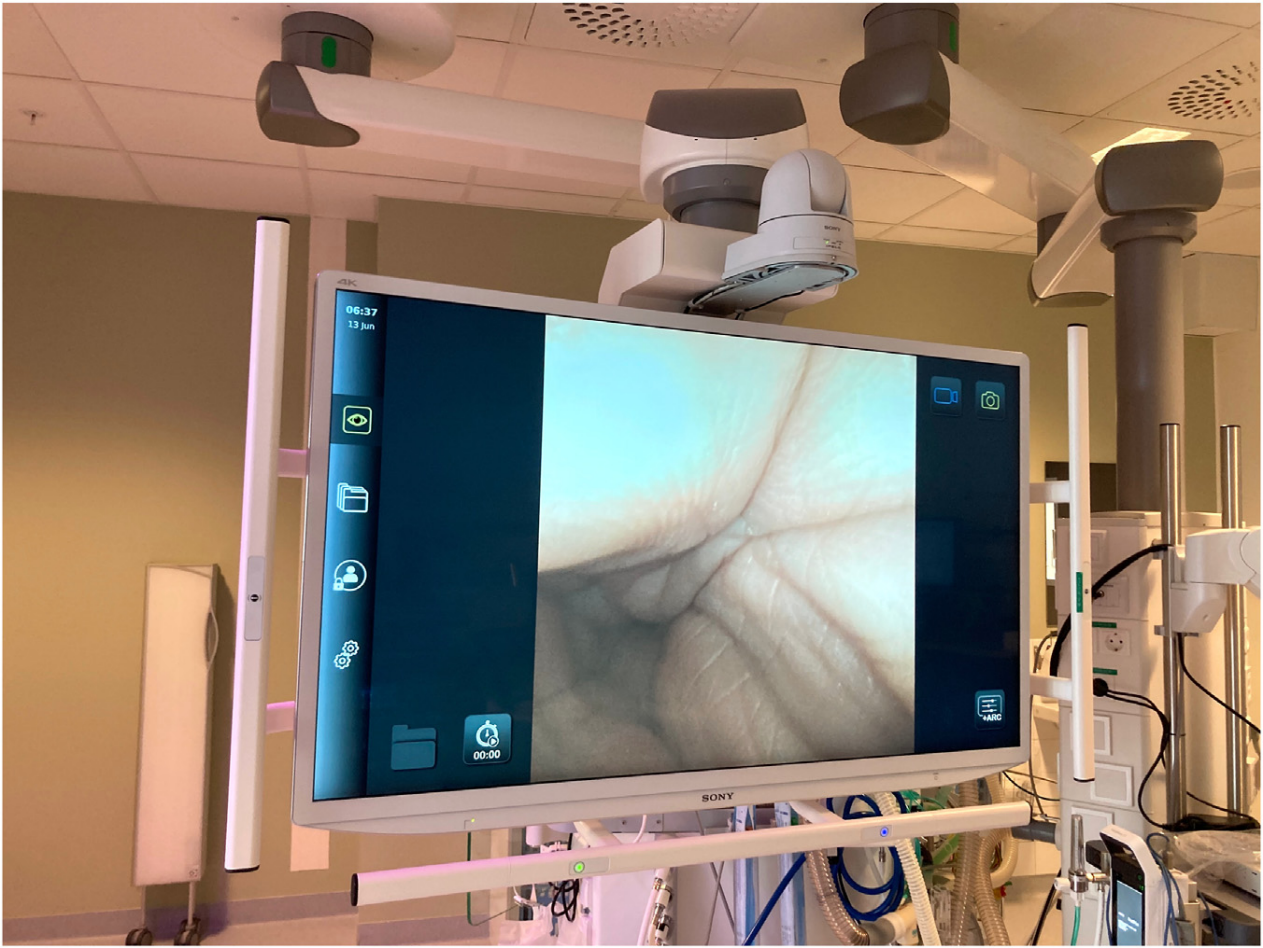
The system can be easily transported and connected to conventional endoscopic imaging systems.

The aScope (Ambu, Ballerup, Denmark) is a recently launched sterile single-use gastroscope. It has a distal diameter of 9.9 mm with dual LEDs, a 2.8-mm working channel, and a water jet. Like in conventional endoscopes, there are 2 wheels and locks and 4 electronic switches with different functions. Function configuration can be done using the touch-screen display. The available functions include photo, video, virtual chromoendoscopy, stopwatch, and timestamp. The scope has a connector that is compatible with standard ancillary devices and tube sets and connects to a small processor, light source, and a screen. The video processor has DVI-D and 3G-SDI video outputs. The system can be easily transported and connected to conventional endoscopic imaging systems (Figs. 1 and 2).

Single-use endoscopes have been used in diagnostic procedures, but their use in therapeutic endoscopic procedures had not been tested. Herein, we present the use of single-use gastroscopes in advanced therapeutic procedures.

## Clinical Cases

### Case 1

**Figure 3:**
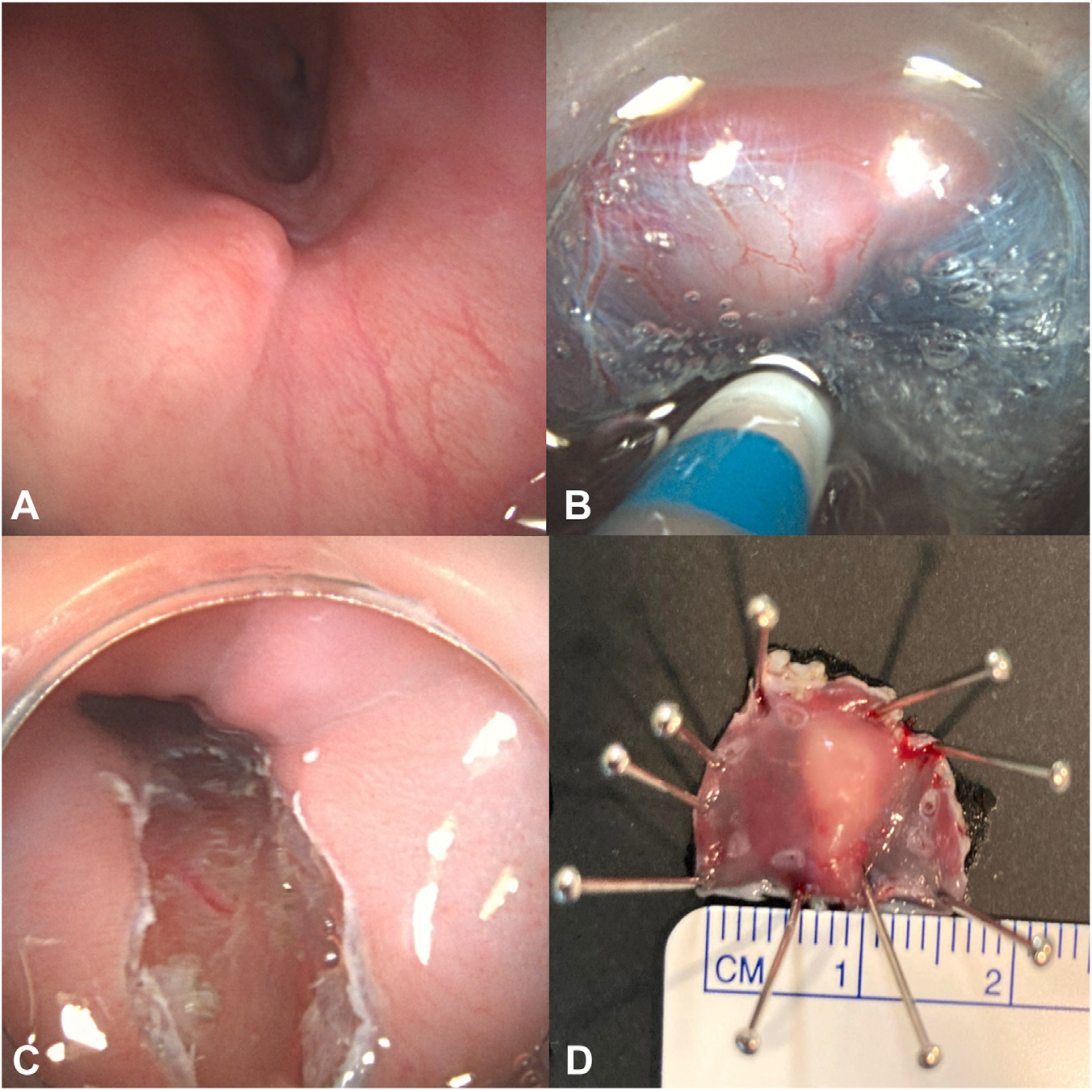
**A,** A 15-mm subepithelial lesion in the distal esophagus. The findings were suggestive of granular cell tumor, but biopsies and EUS were inconclusive. **B,** Endoscopic submucosal dissection (ESD) using an ESD knife. **C,** Mucosal defect after ESD. **D,** ESD specimen that is 15 × 18 mm in size.

A 56-year-old female patient with GERD underwent an EGD that revealed a subepithelial lesion in the distal esophagus. The findings were suggestive of granular cell tumor, but biopsies and EUS were inconclusive. This case was discussed in a multidisciplinary conference, and the decision to perform endoscopic submucosal dissection (ESD) was made. The procedure was performed in the endoscopy unit with the patient under general anesthesia. The ESD was performed using a Dualknife J 1.5 mm (Olympus, Hamburg, Germany). The lesion was resected en bloc after 37 minutes. The specimen was 15 × 18 mm in size, and the pathology analysis revealed an esophageal leiomyoma (Fig. 3). The patient was discharged on the same day. The 4-month follow-up was normal.

### Case 2

**Figure 4:**
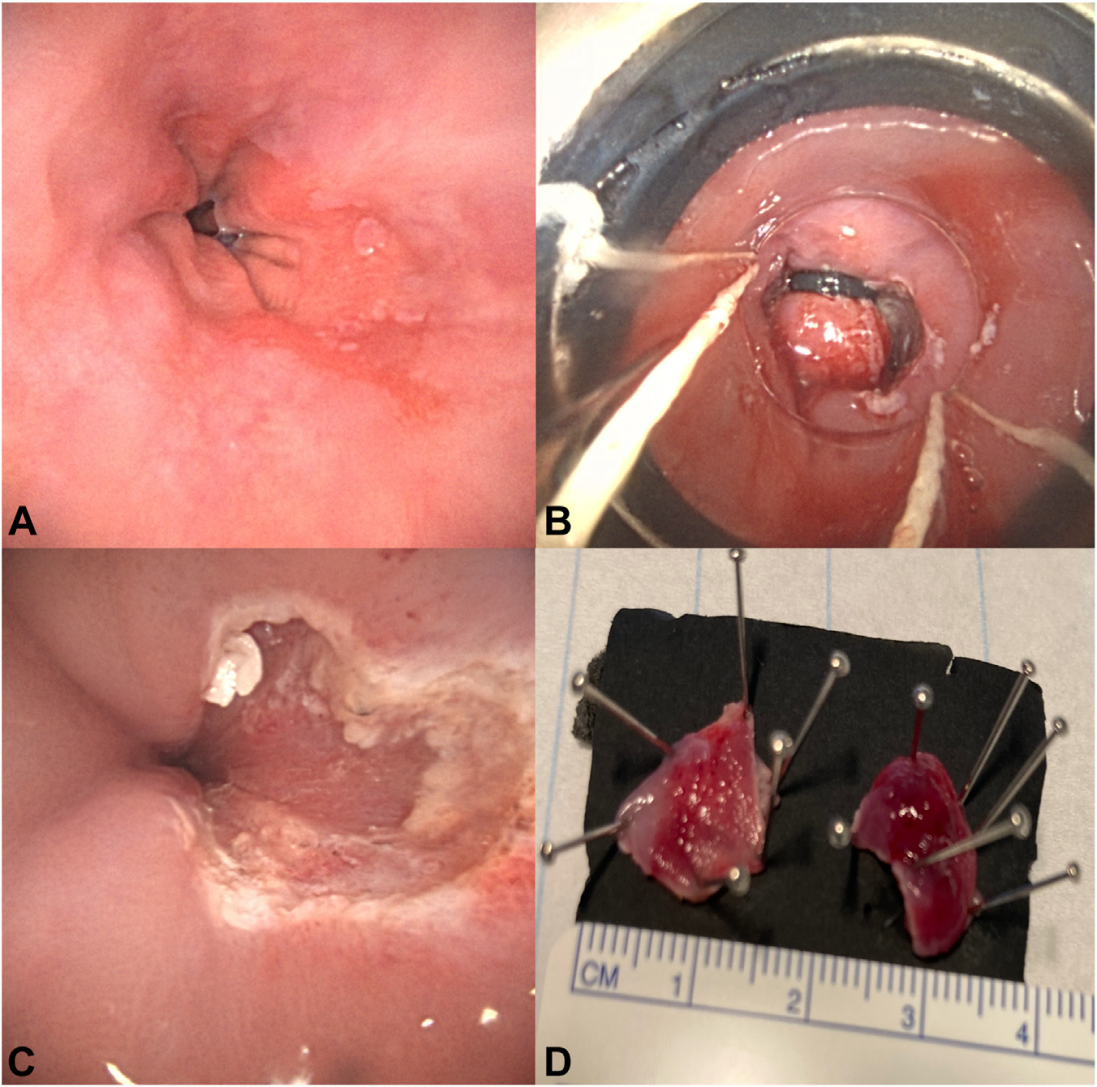
**A,** Barrett’s esophagus (C0M2) with white light; biopsy specimens were taken, and the pathology examination showed intestinal metaplasia and suspicion of rhabdomyoma. **B,** EMR with the Duette system (Cook Medical, Limerick, Ireland). **C,** Mucosal defect after 2 EMRs. **D,** Two specimens obtained.

A 63-year-old male patient with a history of dyspepsia underwent an EGD, and a short Barrett’s esophagus (C0M2) was identified. Biopsy specimens were taken, and the pathology examination showed intestinal metaplasia and suspicion of rhabdomyoma. Owing to this uncommon diagnosis, the case was discussed in a multidisciplinary conference, and a decision for band EMR was made. The procedure was performed with the patient under mild sedation. No lesions were visible, and 2 macrobiopsies were made using a Duette system (Cook Medical, Limerick, Ireland). Pathologic analysis showed Barrett’s esophagus with intestinal metaplasia, low-grade dysplasia, and no signs of rhabdomyoma (Fig. 4).

There were no adverse events during or after both procedures (Video 1, available online at www.videogie.org).

## Conclusion

These new single-use endoscopes were effective in 2 advanced endoscopic procedures. Owing to their safety profile and technical performance, disposable gastroscopes might be useful in different clinic and logistic contexts. These endoscopes appear to be reliable and easy to assemble and use. Compared with conventional endoscopes, which are used and reprocessed several times, single-use endoscopes have no deterioration, so there is consistency in quality and performance at each investigation. Compared with regular gastroscopes, the field of view is shorter, and angulation is 210° (up), 90° (down), 100° (right), and 100° (left). These endoscopes feature digital chromoendoscopy and digital magnification. However, near-focus function is not available. It is not possible to obtain quantitative information from different endoscope providers regarding image quality. It appears that in relation to conventional endoscopes, the image resolution is slightly decreased in the single-use gastroscopes tested. Nevertheless, it was possible to perform advanced endoscopic procedures using these endoscopes.

The use of reusable endoscopes is associated with increased costs and significant environmental impact. Their use might even be economically advantageous at a certain case volume threshold.^10^ Some measures have been developed aiming to reduce their ecologic footprint, and it is likely that more will be developed in the future. In the United States, a take-back system is offered to customers to ensure that plastics and electronics are properly collected, processed, and recycled.

This new field opens the possibility to produce tailored endoscopes in the future, with different characteristics such as length, outer diameter, and working channel. However, further research is required to minimize economic and ecologic barriers related to the use of these devices.

## Disclosure

The authors disclosed no financial relationships relevant to this publication.

## Supplementary data

Video 1 Advanced endoscopic procedures using single-use endoscopes.
